# Current status and influencing factors of family resilience in families of children with epilepsy: a cross-sectional study

**DOI:** 10.3389/fpsyt.2024.1354380

**Published:** 2024-03-07

**Authors:** Wenjing Wei, Lianlian Dong, Jinghua Ye, Zhitian Xiao

**Affiliations:** ^1^ Nursing Department, Sir Run Run Shaw Hospital, Zhejiang University School of Medicine, Hangzhou, China; ^2^ Department of Neurology, China Medical University Shenzhen Children’s Hospital, Shenzhen, China

**Keywords:** children with epilepsy, caregivers, family resilience, social support, depression, influencing factors

## Abstract

**Purpose:**

The study was designed to describe the level of family resilience and identify the protective factors and vulnerability factors of family resilience in families of children with epilepsy. So as to provide theoretical guidance for implementing intervention programs to promote family resilience.

**Methods:**

From November 2020 to July 2021, 258 parents of children with epilepsy were investigated using a convenience sampling method. The questionnaire included demographic data, Chinese-Family Resilience Assessment Scale, Social Support Rating Scale, and the Beck Depression Inventory. SPSS25.0 was used for descriptive statistical analysis, univariate analysis, and multivariate linear regression analysis.

**Results:**

In this study, two hundred and fifty-eight primary caregivers completed the paper questionnaires. The total score of family resilience was (134.97 ± 16.57), which was above the medium level. Multiple linear regression analysis revealed that subjective support (β=0.327, *P*<0.001), comorbidity (β=0.181, *P*<0.05), objective support (β=0.117, *P*<0.05), and parental depression (β=-0.158, *P*<0.05) were significantly related to family resilience. These variables contribute 31.7% of the variance in family resilience (*F*=18.07, *P*< 0.001).

**Conclusion:**

The families of children with epilepsy presented appropriate resilience after the children were diagnosed with epilepsy. Family resilience was correlated with multiple factors, subjective and objective support could be protective factors, comorbidity and parental depression could be vulnerability factors of family resilience. Therefore, future psychosocial interventions could focus on enhancing subjective support and objective support, reducing parental depression, and screening for epilepsy comorbidity to promote the family resilience of children with epilepsy.

## Introduction

Epilepsy is one of the most common neurological chronic disorders in childhood. According to the survey, there are about 50~70 million epilepsy patients worldwide (1, 2). The prevalence of epilepsy in China is about 3.9‰~5.1‰ (3). Although the disease burden declined from 1990 to 2016, epilepsy remains a significant cause of disability and death (4). To be specific, the negative effects of epilepsy extend to the whole family. Children with epilepsy (CWE) are susceptible to combined psychobehavioral problems such as anxiety, depression, attention deficit hyperactivity disorder, et al. (5, 6), with a lower quality of life and a higher risk for death compared to normal children (7, 8). Parents often serve as the primary caregivers in China, which face multiple challenges, such as high medical costs, stigma, anxiety, and depression (9–11), as well as education, employment, and the marriage of children also trouble them. According to the family systems theory (12), the family, as a basic emotional unit, can be affected by the individual’s emotional disorders, which ultimately affects the functioning of the entire family. The diagnose and the long-term rehabilitation process of epilepsy also interfere with family routines and social interactions, leading to decreased quality of parent-child relationships, marital breakdown, and family dysfunction (13). Importantly, family dysfunction might affect the psychological development and health outcomes of CWE (14). Therefore, it is essential to promote family adaptation when facing adversity.

Family resilience, which was proposed by McCubbin (15) in the 1980s. As research has progressed, different theories of family resilience have emerged. The definition of family resilience varies according to the different theories. In the study, family resilience refers to the ability to rebound from adversity and become stronger and more resourceful, which involves shared family belief systems, organizational patterns, and communication processes (16). Families with high resilience are characterized by positive family relationships, less family conflict, and fewer psycho-behavioral problems (17). In addition, family resilience can alleviate caregiver burden in caring for a child with epilepsy and promote psychological adaptation of children with chronic diseases (18, 19). Despite advances in research on family resilience, to our knowledge, research on the family resilience status quo remains limited. Only a few studies assessed family resilience based on small sample sizes. Therefore, explore family resilience among primary caregivers of CWE is critical for healthcare professionals to provide tailored strategies.

The Family Resilience Model proposed by Patterson (20) and previous empirical researches (21–23) in different populations highlights that risk factors (i.e. sociodemographic data, parental depression) and protective factors (i.e. family resources, social support) interactions could influence the resilience of families. Firstly, the effect of demographic data on family resilience is still contradictive. Prior research indicated that the gender and age of caregiver were vital factors in predicting family resilience (24). However, in another study by Dong et al. (19), researchers found that parents’ employment status, medical insurance, duration, and household monthly income were related to the family resilience rather than the gender and age of caregivers. Therefore, it is necessary to further explore the relationship between socio-demographic characteristics and family resilience.

Social support, as an important external resource, refers to emotional, informational, or tangible support from medical staff, caregivers, and non-professional organizations (25). Based on Xiao (26), social support can be categorized into three dimensions, subjective support, objective support, and support utilization. Previous study have found that subjective support could be a protective factor for family resilience in caregivers of children with autism spectrum disorder (27) and stroke patients (28). The support utilization was also been shown to be associated with family resilience (28). However, the role of objective support for family resilience remains unclear. The relationship between the types of social support and family resilience deserves further study among parents with CWE. In addition, some scholars indicated that parental depression may be a risk factor for family resilience in other populations, such as parents of children with cancer (23), families of children with Down syndrome (22), and parents of children with spina bifida (21), which has not been validated in families of CWE. Due to limited reports and cultural differences, it is difficult to generalize the influencing factors of family resilience among CWE in China.

In response to the limitations of previous studies, there is a growing necessity for further study to better understand family resilience among parents of children with epilepsy (especially in China). Therefore, the primary purpose of this study was to identify associated predictors in families of CWE. The present study was designed to answer the following two questions: (1) What level of family resilience was among families of CWE in China? (2) Which of the protective factors and vulnerability factors were associated with family resilience?

## Methods

### Design and data collection

This cross-sectional study was carried out in a tertiary hospital in Guangdong Province. 258 parents of CWE in the neurology ward and neurology outpatient were recruited by convenience sample between November 2020 and July 2021. All participants completed independent questionnaires after obtaining written informed consent. Before collecting data, researchers in this study had received training sessions about data collection procedures, checking, and importing data.

Parents of children with epilepsy were eligible for inclusion in the study if they were (1) aged ≥18 years. (2) mothers or fathers of CWE and primary caregiver (assuming the primary responsibility for caregiving the child, living with and taking care of the child for at least 72 hours per week, or at least 12 hours per day). (3) having a child aged 0~14 years who had been diagnosed with epilepsy by a neurologist in accordance with the International League Against Epilepsy (ILAE) criteria (29). The exclusion criteria for participants were: (1) the child was combined with other complications. (2) the parents were suffering from cognitive impairment or mental illness, and (3) the family suffered several traumatic events in the past half-year, including serious natural disasters, accidents, and sudden deaths of relatives. Informed consent forms were signed by all participants.

In this study, the sample size was calculated using *N*=4Uα^2^S^2^/δ^2^ (30) α=0.05, Uα=1.96, δ = (0.25S, 0.50S) (31). Qiu et al. (32) surveyed 236 parents of chronically ill children using the Family Resilience Assessment Scale, which showed the mean score for family resilience was 127.82 with a standard deviation of 9.942. According to the formula and taking into account a 10% non-response rate, the total sample size ranged from 67 to 270. A total of 286 parents of CWE were invited to participate in the study. Among them, eighteen caregivers declined to complete the questionnaire, and ten participants were excluded because of the incomplete data. Therefore, 258 (96.27%) participants completed the validated questionnaire.

## Measures

### Dependent variable

The Chinese version of Family Resilience Assessment Scale(C-FRAS)translated by Dong et al. (33), was used to assess the level of family resilience. C-FRAS contains four dimensions and 44 items, including family communication and problem solving (FCPS), utilizing social and economic resources (USR), maintaining a positive outlook (MPO), and the ability to make meaning of adversity (AMMA). Each item is rated with a four-point Likert scale ranging from strongly disagree to strongly agree (1-4), with a total score from 44 to 176. The higher the score, the higher the level of family resilience. The Cronbach’s α of C-FRAS was 0.960 (33). In this study, the Cronbach’s α was 0.958, 0.946, 0.888, 0.884, and 0.807 for C-FRAS, FCPS, USR, MPO, and AMMA.

### Independent variables

The sociodemographic data included children’s gender, age, duration of epilepsy, comorbidity, and ketogenic diet. In addition, the general data of primary caregivers (their relationship with the child, age, residence, occupation, monthly family income, education, and religion) was gathered.

Social support was assessed via the Social Support Rating Scale (SSRS) (26), which was used to assess the level of support received from friends, relatives, and healthcare providers. SSRS includes 10 items and three factors (objective support, subjective support, and support utilization). Among them, the scores for items 5, 6, and 7 are based on the number of choices, and other items are scored on a four-point Likert scale. The higher marks represent higher levels of social support received by the individual. The Cronbach’s α was 0.707 in this study.

The parents’ depressive symptoms was assessed using the Beck Depression Inventory (BDI) (34). It consists of 21 items, each item is scored from 0 to 3, with a total score ranging from 0 to 63. Higher scores represent the increasing severity of depressive symptoms. The Cronbach’s α in the present study was 0.849.

### Statistical analysis

All data were imported into EpiData 3.1. IBM SPSS Statistics (version 25.0, IBM Corp, Armonk, NY, USA) was used for statistical analysis. Descriptive statistics were presented as Mean ± standard deviation (M ± SD) (or median, interquartile range (IQR)), frequency (N) and percentage (%). Independent sample *t*-test and one-way analyses of variance with Scheffé’s *post-hoc* comparisons were performed to test the relation between demographic factors and family resilience. Simultaneously, normality and homogeneity of variance tests were conducted. Pearson correlation analysis was applied to examine the relationship among social support, parents’ depressive symptoms, and family resilience. After that, multiple linear regression analysis was performed to explore the main influencing factors of family resilience. Specifically, the total score of family resilience was the dependent variable, and variables that were statistically significant in the univariate and correlation analyses were the independent variables for multiple stepwise regression analysis. The variance inflation factor (VIF) was applied to examine the multicollinearity between the independent variables. For unordered multi-categorical, dummy variable assignment was used. Two-sided p-value< 0.05 was statistically significant.

## Results

### Descriptive statistics

#### Demographic characteristics

As shown in **Table 1**. Children with epilepsy had a mean age of 5.80 ± 3.86 years, with the median disease duration being 24 months (IQR 10-48). Among 258 parents of children with epilepsy, 204 (79.10%) were mothers, and 54 (20.90%) were fathers. Participants’ ages ranged from 23 to 48 years, and the mean age was 35.44 ± 5.03 years.

**Table 1:** Descriptive statistics for sociodemographic characteristics (N=258).

Variable	Categories	N(%)	C-FRAS total ( $\bar{x}\pm s$ )	*F/t*	*P*
Child gender	Male	148(57.4)	133.86 ± 16.25	-1.255	0.211
	Female	110(42.6)	136.47 ± 16.96		
Age of children (Years)	≤3	90(34.9)	131.03 ± 17.14^c^	4.033	0.019
	4∼6	59(22.9)	136.63 ± 18.10		
	7∼14	109(42.2)	137.33 ± 14.68		
Time since diagnosis (Age at first diagnosis(years)	0∼1	112(43.4)	131.45 ± 17.72 ^b^	4.609	0.011
	2∼6	98(38.0)	137.74 ± 14.85		
	7∼14	48(18.6)	137.54 ± 15.90		
Duration(Years)	≤1	77(29.8)	134.30 ± 16.21	0.149	0.861
	1∼3	87(33.7)	135.70 ± 17.38		
	>3	94(36.4)	134.85 ± 16.25		
Comorbidity	Yes	56(21.7)	125.29 ± 14.13	-5.607	<0.001
	No	202(78.3)	137.66 ± 16.22		
Ketogenic diet	Yes	36(14.0)	127.94 ± 15.24	-2.779	0.006
	No	222(86.0)	136.11 ± 16.53		
Principal caregiver	Father	54(20.9)	137.24 ± 15.35	-1.132	0.259
	Mother	204(79.1)	134.37 ± 16.87		
Occupation	Employed	154(59.7)	135.45 ± 15.50	0.551	0.582
	Unemployed	104(40.3)	134.26 ± 18.09		
Religion	Yes	26(10.1)	141.50 ± 16.78	2.132	0.034
	No	232(89.9)	134.24 ± 16.42		
Education	High school or below	93(36.0)	134.51 ± 17.00	1.321	0.268
	College	76(29.5)	132.61 ± 16.43		
	Undergraduate	79(30.6)	137.15 ± 16.43		
	Graduate or above	10(3.9)	140.10 ± 13.39		
Monthly family income (Yuan)	<5000	27(10.5)	126.33 ± 14.80 ^c, d^	3.670	0.013
	5000∼10000	73(28.3)	133.47 ± 16.43		
	10000∼15000	56(21.7)	137.39 ± 16.70		
	>15000	102(39.5)	137.01 ± 16.40		
Residence	Countryside	49(19.0)	128.63 ± 15.50 ^c^	5.799	0.003
	Suburban	29(11.2)	132.10 ± 13.07		
	City	180(69.8)	137.16 ± 16.91		

C-FRAS, Chinese-Family Resilience Assessment Scale. Post-hoc tests a: compare with Layer 2 P<0.05; c:compare with Layer 3, P<0.05; d: compare with Layer 4, P<0.05.

Descriptive statistics for family resilience, social support, and depression were shown in **Table 2**. The mean score of family resilience was 134.97 ± 16.57, family communication and problem solving had the highest scores, while utilizing social and economic resources scored the lowest.

**Table 2:** Descriptive statistics for family resilience, social support and depression (N=258).

Variables	Minimum	Maximum	M ± SD	Mean item score
Family resilience	95	176	134.97 ± 16.57	3.07 ± 0.38
FCPS	57	108	85.70 ± 11.08	3.17 ± 0.41
AMMA	6	12	9.29 ± 1.24	3.10 ± 0.41
MPO	10	24	18.25 ± 3.03	3.04 ± 0.51
USR	13	32	21.72 ± 3.48	2.71 ± 0.44
Social support	19	56	38.70 ± 6.04	3.87 ± 0.60
Objective support	3	19	10.21 ± 2.52	3.40 ± 0.84
Subjective support	11	32	21.71 ± 3.93	5.43 ± 0.98
Utilization of Support	3	12	6.78 ± 1.67	2.26 ± 0.56
Depression	0	47	11.00 ± 9.18	0.52 ± 0.44

FCPS, Family Communication and Problem Solving; USR, Utilizing Social and economic Resources; MPO, Maintaining a Positive Outlook; AMMA, Ability to Make Meaning of Adversity.

#### Univariate analysis

Univariate analysis was used to analyze the factors related to family resilience. Results in the **Table 1** showed that the age of children (*F*=4.033 *P*=0.019), time since diagnosis (*F*=4.609 *P*=0.011), comorbidity (*t*=-5.607 *P*<0.001), ketogenic diet (*t*=-2.779 *P*=0.006), religion of the primary caregiver (*t*=2.132 *P*=0.034), monthly family income (*F*=3.670 *P*=0.013), and residence (*F*=5,799 *P*=0.003) were statistically significant related to the C-FRAS.

*Post-hoc* analyses showed a statistically significant difference of the C-FRAS scores in CWE aged 0~3 years and 7~14years (*P*<0.05). To be specific, CWE aged 0 to 3 years had lower levels of family resilience in comparison to another group. In addition, parents of CWE had a lower family resilience level when the child was diagnosed within 1 year than when it was 2~6 years. Results in **Table 1** indicated that there was a statistically significant difference in the C-FRAS score in the monthly family income of less than 5000 vs 10000~15000 (*P*<0.05) and 0~5000 vs more than 15000 (*P*<0.05). In terms of residence, we found that the C-FRAS score in families of CWE living in the countryside had lower levels than did those living in the city.

#### Correlations between family resilience, social support and depression

The results of correlation analysis showed significant correlations among these variables. Family resilience was positively associated with social support (*r*=0.478,*P*<0.01) and negatively related to psychological distress (*r*=-0.389, *P*<0.01). The bivariate correlations indicate that the following multiple linear regression analysis can be carried out, as presented in **Table 3**.

**Table 3:** Correlation of main variables among parents of CWE.

Variables	C-FRAS	FCPS	USR	MPO	MM
Social support	0.478^**^	0.443^**^	0.411^**^	0.388^**^	0.333^**^
Objective support	0.248^**^	0.253^**^	0.151^*^	0.198^**^	0.155^*^
Subjective support	0.473^**^	0.426^**^	0.434^**^	0.398^**^	0.318^**^
Utilization of Support	0.242^**^	0.217^**^	0.237^**^	0.167^**^	0.224^**^
Depression	-0.389^**^	-0.376^**^	-0.295^**^	-0.298^**^	-0.282^**^

C-FRAS, Chinese-Family Resilience Assessment Scale; FCPS, Family Communication and Problem Solving; USR, Utilizing Social and economic Resources; MPO, Maintaining a Positive Outlook; AMMA, Ability to Make Meaning of Adversity. **p<0.01 *p<0.05.

#### Multiple stepwise regression analysis

In order to further understand the influences of various variables on the resilience of children with epilepsy. Family resilience was the dependent variable. Sociodemographic variables that were significantly associated with family resilience, social support, and depression were selected as independent variables for the multiple stepwise regression analysis. Dummy variable assignment for residence and the assignment method of independent variables was shown in **Table 4**. The results showed that the variance inflation factor ranged between 1.116 and 1.383, which were all less than 5, indicating that there was no multicollinearity between the independent variables (35). The influencing factors of family resilience in families of CWE included subjective support, depression, objective support and comorbidity. These factors accounted for 31.7% of the variance in family resilience (*F*=18.07, *P*< 0.001). The result of the multivariate analysis demonstrated in **Table 5**.

**Table 4:** Assignment method and code of independent variables.

Independent variable	Assignment method	Code
Age of children	0∼3 = 1,4∼6 = 2	X1
(years)	7∼14 = 3	
Time since diagnosis	0∼1 = 1, 2∼6 = 2	X2
(years)	7∼14 = 3	
Comorbidity	Yes=1, No=2	X3
ketogenic diet	Yes=1, No=2	X4
Religion	Yes=1, No=2	X5
Monthly family income (Yuan)	<5000 = 1	X6
	5000∼10000 = 2	
	10000∼15000 = 3	
	>15000 = 4	
Residence	Countryside Suburban City	X7 = 0 X8 = 0 X7 = 1 X8 = 0 X7 = 0 X8 = 1
Objective Support	Actual score	X9
Subjective support	Actual score	X10
Utilization of Support	Actual score	X11
Depression	Actual score	X12

**Table 5:** Multiple stepwise regression of influencing factors associated with family resilience.

Variables	*B*	*SE*	*β*	*t*	*P*	VIF
Constant	94.375	9.831	–	9.599	<0.001	
Subjective support	1.378	0.245	0.327	5.625	<0.001	1.271
Depression	-0.285	0.109	-0.158	-2.611	0.01	1.383
Comorbidity	7.279	2.192	0.181	3.320	0.001	1.124
Objective support	0.768	0.360	0.117	2.142	0.033	1.116

R=0.580, R^2 = ^0.336, adjusted R^2 = ^0.317.

## Discussion

In this study, we assessed the level of family resilience and identified influencing factors that might predict family resilience, enhancing our understanding for resilience in families of children with epilepsy. The results from the C-FRAS showed that parents experienced some positive changes after the children were diagnosed with epilepsy. In addition, subjective support, comorbidity, objective support, and parental depression were demonstrated as independent influencing factors of family resilience.

The results from the FRAS showed that the mean score of family resilience was 134.97(SD=16.57) and the average item score was 3.07(SD=0.38), which was consistent with Liu et al. (36) with the average score was 3.06(SD=0.28). The results indicated a relatively high level of family resilience among children with epilepsy. Among the dimensions of family resilience for parents of CWE, the highest mean scores were family communication and problem solving and the lowest was utilizing social and economic resources, which was consistent with Shi’s study in children with acute leukemia (37). This may be explained by the cultural misconception towards epilepsy results in epilepsy stigma. To be honest, there are still negative attitudes and misconceptions towards epilepsy in China, especially in rural areas, where epilepsy is seen as a kind of infectious disease or mental illness (38), which limits the parents seeking assistance from the friends and community. This is considered the greatest handicap for the utilization of socio-economic resources. This findings suggest that interventions on improving the utilization of social resources would be essential to promote the family resilience of CWE. In another light, it is imperative to strengthen multi-faceted health education in China among parents of CWE, patients, and the public to improve their understanding towards epilepsy.

Our finding revealed that social support was positively associated with family resilience, with subjective support was the most vital factor with an important ratio of 22% in predicting family resilience of CWE, in line with Jadiri’s research in children with autism spectrum disorder (27). This could be attributed to the emotional support from the family and community could alleviate parents’ helplessness and isolation in the face of adversity, thereby enhancing the family’s ability to resist risks. This finding highlights that nurses and family members could provide emotional support to parents of CWE and encourage caregivers to communicate with others. Meanwhile, peer support among parents of CWE could also be carried out in the form of face-to-face or WeChat to promote information exchange and sharing of caregiving experiences, which further enhance the caregivers’ subjective sense of well-being and family resilience (39).

One of our important findings was that parental depression was a negative predictor of family resilience. That is, depressive symptoms in parents of CWE could significantly influence family resilience. This result was consistent with that seen in previous studies (21, 36). This may be explained by the fact that high medical expenses, unpredictability of seizures, and limited family social interaction among parents of CWE would increase the risk for depression compared with parents of healthy children (11). Moreover, this psychological distress was detrimental to the health-related quality of life of family members and family system (40), which was not conducive to the family resilience. However, when the levels of family resilience was high, it could promote the mobilization of social resources, which further reduced family members’ negative emotions and improved parental mental health (41). Given that the parents’ depression was a predictor for family resilience, this finding highlights the importance of screening the psychological distress and developing interventions to reduce depression among parents of CWE.

In accordance with Wei et al. (42), objective support was proven to be one of the protective factors of family resilience. In this study, support utilization did not enter the regression analyze in this study, this may be attributed to the fact that its intrinsic impact on family resilience is weaker than subjective support and objective support. Objective support refers to the actual social support received, including direct material help and participation in social networks and group relationships, which is visible support (26). In other words, the more objective support received by families of children with epilepsy, the higher level of family resilience. A possible explanation for this is that frequent hospitalization and rehabilitation of CWE places a heavy financial burden on the family, while objective support can alleviate the financial and caregiving burden of parents, providing a basis for rebuilding family resilience (43). Therefore, the current study suggests that measures should be taken by governments and policymakers to increase policy coverage, incorporate more epilepsy medications into the coverage of health insurance, and establish special funds for families of children with epilepsy to reduce the financial burden, as well as to improve family resilience.

According to the results of this study, comorbidity was found as an important factor related to family resilience. Compared to children without comorbidity, CWE with comorbidity got lower scores of family resilience, which meant a low level of family resilience. On one hand, epilepsy comorbidity is associated with increased neurology outpatient visits, emergency department visits, and hospitalizations, which further increase the health resource utilization and financial burden on the family (44). On the other hand, primary caregivers of CWE who have comorbidity perceive higher levels of stress and stigma compared to CWE without comorbidity (9). The above reasons explain that caring for a child with epilepsy comorbidity places an enormous psychological and financial burden on the parents, which is detrimental to the recovery of family functioning and family adaptation. Therefore, our result suggests that healthcare providers should value epilepsy comorbidity, early screening, diagnosis, and treatment for these comorbidities to improve the long-term prognosis and family functioning.

## Limitations and recommendations for future studies

There are several limitations in the present study. Firstly, we are unable to speculate the dynamic changes among variables and parents’ feelings towards family resilience owing to the nature of the cross-sectional study. In the future, longitudinal and qualitative studies can be carried out to further understand the trajectories of family resilience. Secondly, this study was conducted only from one tertiary hospital in Guangdong Province, which has a limited sample representative. Future study can conduct a multi-center survey to enrich factors in predicting family resilience. Finally, causal relationships among variables cannot be confirmed by multiple stepwise regression analysis alone, prospective research can be carried out in the future.

## Conclusion

This cross-sectional study explored the level of family resilience for primary caregivers of CWE and associated influencing factors. The results showed that the family resilience of CWE was above the medium level. Subjective support, comorbidity, objective support, and parents’ depression were significantly related to family resilience. Among these influencing factors, subjective and objective support were protective factors, epilepsy comorbidity and parental depression could be vulnerability factors of family resilience. This finding hints that healthcare professionals should consider protective and risk factors of family resilience when providing family-centered care to children with epilepsy, and develop interventions that promote protective factors and reduce risk factors, which ultimately contribute to family resilience.

## Data availability statement

The original contributions presented in the study are included in the article/Supplementary Material. Further inquiries can be directed to the corresponding author.

## Ethics statement

The studies involving humans were approved by Shenzhen Children’s Hospital (No.2020067). The studies were conducted in accordance with the local legislation and institutional requirements. The participants provided their written informed consent to participate in this study.

## Author contributions

WW: Data curation, Formal Analysis, Investigation, Writing – original draft, Writing – review & editing. LD: Supervision, Writing – review & editing, Validation. JY: Data curation, Investigation, Resources, Writing – review & editing. ZX: Data curation, Funding acquisition, Investigation, Methodology, Resources, Writing – review & editing.
